# Modern management of locally advanced urothelial carcinoma of the upper urinary tract

**DOI:** 10.1007/s00345-026-06521-y

**Published:** 2026-07-21

**Authors:** Yanchun Ma, Maximilian Postel, Michael Karl Melzer, Christian Bolenz, Friedemann Zengerling

**Affiliations:** Department of Urology and Pediatric Urology, University Hospital Ulm, Ulm, Germany

**Keywords:** locally advanced UTUC, upper tract urothelial carcinoma, systemic therapy, clinical trials, novel therapy strategies, treatment advances

## Abstract

**Purpose:**

Upper tract urothelial carcinoma (UTUC) is a rare malignancy arising from the renal pelvis or ureter and is often diagnosed at an advanced stage. Prognosis depends strongly on tumor stage and grade, but remains poorer than that of bladder urothelial carcinoma.

**Methods:**

This review article summarizes current evidence on the management of locally advanced but resectable UTUC, focusing on perioperative systemic therapy, clinical guideline recommendations, and emerging therapeutic strategies.

**Results:**

In high risk cases, radical nephroureterectomy (RNU) with bladder cuff excision is the surgical state of the art. To reduce relapse rates, perioperative systemic therapy is gaining increasing importance. Neoadjuvant platinum-based chemotherapy (+/- durvalumab) has shown promising rates of pathological downstaging, however, high level evidence is still lacking. In contrast, evidence for adjuvant therapy is stronger. Adjuvant platinum-based chemotherapy should be offered to patients with pT2-pT4 or pN+ disease within 90 days after RNU. Immune checkpoint inhibitors show activity in perioperative settings, but potential benefits in UTUC subgroups remain unclear. There are ongoing trials combining immunotherapy, chemotherapy, or targeted agents. Molecular profiling and novel strategies, such as mRNA vaccines and antibody-drug conjugates, may enable more personalized approaches and reshape the therapeutic landscape of UTUC.

**Conclusion:**

Management of locally advanced but resectable UTUC is evolving rapidly, driven by advances in perioperative systemic therapies and a growing understanding of the disease’s molecular biology. However, prognosis remains poor, underscoring the need to further improve treatment options.

## Introduction

Upper tract urothelial carcinoma (UTUC) are tumors of urothelial origin that arise in the renal pelvis or ureter. Compared to urothelial carcinoma of bladder (BUC), also called lower tract urothelial carcinoma (LTUC), UTUC is a rare malignancy, accounting for only 5–10% of all urothelial malignancies [1]. At the time of initial diagnosis, UTUC is frequently diagnosed at an advanced stage (Table 1). Nevertheless, it should be considered, that frequencies are difficult to compare as coding of tumor stage may vary and moreover, there is a reporting bias for the frequency of advanced stages. The prognosis for UTUC is generally poorer and highly dependent on tumor stage and grade [1].

**Table 1:** T- and N-stadium at diagnosis: UTUC vs. LTUC

Stage at diagnosis	UTUC (% at diagnosis) [2–6]	LTUC (% at diagnosis) [7, 8]
Non-muscle invasive (Tis/Ta/T1)	~ 30–50	~ 75–85
Muscle invasive (≥ T2)	~ 50–70	~ 15–25
Node positive (N+)	~ 5–20	~ 5–7

UTUC: upper tract urothelial carcinoma; LTUC: lower tract urothelial carcinoma

Despite its presumed similarities to BUC, UTUC requires a distinct clinical management and therapeutic approach due to its different anatomical (e.g. thinner muscularis propria layer) and molecular characteristics (e.g. higher alteration rates for FGFR3, HRAS and CDKN2B) [9]. However, the low incidence of the disease and the lack of understanding of its molecular pathogenesis pose significant challenges to the optimization of treatment strategies of UTUC.

This article provides an overview of current guideline recommendations and recent advances in the management of locally advanced but resectable UTUC (defined as ≥T2 and/or N + and M0 tumors), with a focus on the neoadjuvant and adjuvant therapeutic strategies.

## Surgery

In cases of high-risk, organ-confined UTUC, radical nephroureterectomy (RNU) with en bloc removal of the kidney and ureter, including ipsilateral bladder cuff excision constitutes the surgical gold standard. RNU can be performed either through an open surgical approach or via minimally invasive techniques (laparoscopic or robot-assisted). Minimally invasive approaches are associated with shorter hospital stays and, importantly, demonstrate oncological outcomes and complication rates comparable to those of open surgery rates [10, 11]. However, robot-assisted RNU has been reported to be associated with a lower intravesical recurrence-free survival compared with open RNU (HR 1.73; 95% CI 1.22–2.45) [12].

Although high-level evidence supporting lymphadenectomy (LND) in UTUC is lacking, current guidelines propose its use in high-risk disease [1, 13]. Reported rates of nodal involvement in UTUC range from 14% to 40%, depending on patient selection and the extent of LND [14–17]. A study based on the SEER database (*n* = 7,278) evaluating the survival benefit of LND in patients undergoing RNU demonstrated a significant improvement in overall survival (OS) among patients with T3 (adjusted HR: 0.88; 95% CI 0.78–0.99) and T4 disease (adjusted HR: 0.74; 95% CI 0.59–0.94) [18]. Similarly, cancer-specific survival (CSS) was significantly improved in patients with T3 (adjusted HR 0.83; 95% CI 0.73–0.98) and T4 tumors (adjusted HR 0.64; 95% CI 0.47–0.88), whereas no statistically significant differences in OS or CSS were observed in patients with ≤T2 tumors [18]. A comprehensive review of retrospective data showed a beneficial effect of LND in ≥pT2 UTUC tumours in terms of CSS and local recurrence, especially in advanced tumors of the renal pelvis, whereas the role of LND for ureteral tumors remained unclear [19].

Following RNU, bladder tumor recurrence occurs in approximately 22–47% of patients. To reduce the risk, a single intravesical instillation of chemotherapy such as mitomycin C is recommended within 2 to 10 days postoperatively. This approach yields an absolute risk reduction of about 11% and a relative risk reduction of around 40% for intravesical recurrence [20]. Current evidence indicates that single postoperative instillation is sufficient, as increasing the number of instillations did not demonstrate any significant benefit in lowering recurrence rates [21]. Recently, gemcitabine has also increasingly been used as an intravesical instillation agent. Although gemcitabine has shown good efficacy as a postoperative instillation in the context of BUC, evidence supporting its use following RNU remains limited. Nevertheless, gemcitabine is increasingly favored due to concerns of chemical peritonitis in case of extravesical extravasation associated with alternative agents [22, 23].

## Perioperative systemic therapy

Despite optimal surgical management, relapse rates, particularly for locally advanced UTUC, remain high [24]. Thus, perioperative systemic therapies have gained increasing attention in recent years for their potential to improve oncological outcomes. As UTUC patients often contribute a small proportion in perioperative urothelial carcinoma (UC) phase III trials or are even excluded completely, the results of these clinical trials are to interpreted with caution, as they should not be extrapolated to UTUC in general .

## Neoadjuvant chemotherapy

The rationale for neoadjuvant chemotherapy (NAC) is to target micrometastases and achieve tumor downstaging, ideally resulting in a pathological complete response (pCR) and improved resectability of locally advanced tumors. Even though neoadjuvant chemotherapy is an established component of treatment for muscle-invasive urothelial carcinoma of the bladder (MIBC), data from randomized clinical trials for UTUC remains lacking. However, several retrospective studies have reported favorable pathological downstaging and survival rates with NAC compared to RNU alone. In a prospective phase II trial, Margulis et al. evaluated accelerated methotrexate, vinblastine, doxorubicin, and cisplatin (aMVAC) in patients with high grade UTUC and a baseline GFR > 50 ml/min. Patients with a GFR of 30–50 ml/min were assigned to a gemcitabine/carboplatin arm, which was closed early due to low accrual (*n* = 6). Four cycles of NAC were planned in total. In the aMVAC cohort, 30 patients were enrolled, of whom 24 completed all four cycles. Preoperative imaging showed no disease progression and no treatment-related deaths occurred. A pCR rate of 13.8% and a final pathological stage ≤ypT1 in 60% of patients undergoing RNU were reported. However, median relapse-free survival following aMVAC and RNU was not reached at a median follow-up of 21.1 months [25]. 

Similarly, a multicenter prospective phase II trial including 57 cisplatin-eligible patients (eGFR ≥ 55 mL/min) with high-risk localized UTUC evaluated NAC using gemcitabine and split-dose cisplatin, based on regimens established in MIBC with favorable tolerability [26]. A total of 89% of patients completed at least three cycles of NAC, early therapy discontinuation was required for 14% of the patients due to toxicities, including thromboembolic events (5%), renal dysfunction (4%), and cardiovascular events (2%). The primary endpoint was achieved with a pathologic response (< ypT2N0) of 63% (95% CI 49–76). In addition, a 19% pCR rate was reported. Two-year and five-year progression free survival (PFS) rates were 89% (95% CI 81–98) and 72% (95% CI 59–87), respectively, while two-year and five-year OS rates were 93% (95% CI 86–100) and 79% (95% CI 67–94), respectively. In both analyses, pathologic response was associated with improved PFS and OS [27]. Updated long-term oncologic outcomes, with a median follow-up of 5.4 years, demonstrasted the estimated 7-years survival rates of 60% for disease-free survival (DFS), 77% for CSS and 72% for OS. In addition, pathologic response was associated with superior long-term oncologic outcomes. Patients who responded to NAC showed significantly better DFS (78% vs. 31%; HR 0.15, 95% CI 0.06–0.39; *p* < 0.001), CSS (90% vs. 56%; HR 0.16, 95% CI 0.04–0.60; *p* = 0.002), and OS (90% vs. 56%; HR 0.18, 95% CI 0.06–0.50; *p* < 0.001) in comparison to non-responders [28]. 

Despite these promising findings, the overall level of evidence remains limited. The absence of randomized phase III trials, small patient cohorts, and heterogeneity across studies preclude definitive conclusions [29]. Consequently, guideline recommendations remain inconsistent. The European Association of Urology (EAU) guidelines do not provide clear recommendations for NAC in UTUC due to the absence of level 1 evidence and challenges in accurate preoperative staging [1, 30]. In contrast, the American Urological Association (AUA) guidelines suggest a cisplatin-based NAC for high-risk UTUC, particularly for those patients at high risk of renal function decline post surgery, based on promising phase II data and extrapolation from high level evidence in MIBC [13].

Incorporating renal function into treatment decision-making is clinically justified, as NAC can be administered when renal function is optimal, thereby increasing the proportion of patients eligible for platinum-based chemotherapy. A retrospective analysis of 388 patients showed that while 49% had an eGFR above 60 mL/min prior to RNU, only 19% maintained this level following surgery [31]. Particularly renal function of elderly patients ≥ 70 years significantly deteriorate following surgery and are therefore more likely to become ineligible for adjuvant chemotherapy (AC) [31, 32]. Hensley et al. established a nomogram for predicting postoperative eGFR, using multivariable predictors that includes diabetes, hypertension, tumor size, age and preoperative eGFR [33].

Despite the advantages of NAC, identifying suitable patient who will most likely benefit from NAC remains critical, especially given the limitations of accurate preoperative staging in UTUC bearing the potential for overtreatment in patients with less advanced disease. In this context, a prospective observational study by Huelster et al. involving 30 patients showed that circulating tumor DNA (ctDNA) could be used to identify muscle-invasive and non-organ-confined UTUC prior to surgery, suggesting a potential role for ctDNA in optimizing patient selection for NAC [34]. However, further evidence is required before ctDNA can be implemented into clinical practice.

## Adjuvant chemotherapy

Unlike NAC, the use of adjuvant chemotherapy (AC) in UTUC is clearly supported by the guidelines and platinum-based chemotherapy should be offered to patients with pT2-pT4 or pN+ UTUC within 90 days following RNU [1, 13]. This recommendation is primarily based on the POUT (Peri-Operative Chemotherapy versus Surveillance in Upper Tract Urothelial Cancer) trial which analysed the efficacy of adjuvant chemotherapy compared to surveillance in patients with either pT2-T4 or pN+ UTUC. In this phase III, open-label, randomised controlled trial, Birtle et al. observed a significant improvement in DFS (HR 0.45; 95% CI 0.30–0.68; *p* = 0.0001) and prolonged metastasis-free survival (HR 0.48; 95% CI 0.31–0.74; *p* = 0.0007) with adjuvant chemotherapy at a median follow-up of 30.3 months compared to the control group [35]. In addition, updated results on prolonged overall survival of 66% versus 57% (HR 0.68; 95% CI 0.46-1.00; *p* = 0.049) and an improved 5-year-DFS of 62% versus 45% in the control group (HR 0.55; 95% CI 0.38–0.80; *p* = 0.001) also favoured the use of AC [36]. Treatment consisted of four cycles of platinum-based chemotherapy in combination with gemcitabine. In patients with impaired renal function (GFR ≥ 30 mL/min and < 50 mL/min), Carboplatin was permitted instead of Cisplatin (Table 2). Subgroup analysis demonstrated a significant DFS benefit for patients receiving gemcitabine/cisplatin (HR 0.53; 95% CI 0.33–0.86), whereas in gemcitabine/carboplatin cohort the upper limit of the 95% CI slightly exceeded 1.0 (HR 0.58; 95% CI 0.33–1.03). Similarily, OS was significantly improved for the gemcitabine/cisplatin subgroup (HR 0.57; 95% CI 0.33–0.97) but not in the carboplatin/gemcitabine subgroup (HR 0.87; 95% CI 0.50–1.53). Consequently, cisplatin should be preferred whenever renal function allows [36]. Regarding tolerability, grade ≥ 3 treatment-related adverse events occurred in 44% of patients receiving chemotherapy compared with 3% in the surveillance group, with no treatment-related deaths. Overall, 75% of patients completed all four cycles, with comparable completion rates between cisplatin- and carboplatin-based regimens (75% vs. 76%) [35]. 

**Table 2:** Eligibility criteria for platinum-based chemotherapy [1]

Category	Criteria
Cisplatin-eligible	- ECOG PS 0–1 and - GFR > 50–60 mL/min and - Audiometric hearing loss grade < 2 and - Peripheral neuropathy grade < 2 and - Cardiac insufficiency NYHA class < III
Carboplatin-eligible (in adjuvant setting)	- ECOG PS 2 or GFR 30–60 mL/min or not fulfilling other cisplatin-eligibility criteria
Platinum-ineligible	Any of the following: - GFR < 30 mL/min - ECOG PS > 2 - ECOG PS 2 and GFR < 60 mL/min - Comorbidities > Grade 2

ECOG PS: Eastern Cooperative Oncology performance status; GFR: glomerular filtration rate

## Immunotherapy

Immunotherapy in the neoadjuvant setting remains the subject of ongoing research and due to limited evidence it is currently not part of the standard of care for UTUC. A small feasibility study (PURE-02) evaluating the efficacy of neoadjuvant pembrolizumab in 10 patients with high-risk UTUC showed no positive therapeutic effect [37]. In contrast, a single-arm, prospective phase II trial of the PD-1 inhibitor tislelizumab in 20 cisplatin-ineligible patients with high-risk UTUC showed a pCR rate of 20% and tumor downstaging to ≤ypT1N0 in 45% of patients, with good tolerability and no significant renal impairment [38]. Another phase II study comprising 17 cisplatin-ineligible patients with high-risk UTUC treated with a dual immunotherapy with nivolumab and ipilimumab showed promising results with a pCR in one third of patients. Interestingly, all six patients with pathogenic germline variants in mismatch repair genes or microsatellite instability (MSI)-high tumors achieved < ypT2pN0 at RNU and remained free of disease, making this selected patient cohort ideal candidates for neoadjuvant dual immunotherapy [39]. The mismatch repair deficiency (dMMR)/MSI-high-phenotype in UTUC is usually associated with Lynch syndrome and is characterized by increased tumor immunogenicity and therefore likely more responsive to immunotherapy than sporadic UTUC [40, 41]. For unresectable or metastatic tumors with MSI-high status or MMR deficiency after failure of prior therapy with no therapy alternatives, the immune checkpoint inhibitor (ICI) pembrolizumab is approved by the Federal Drug Administration (FDA) across tumor types. However, the role of immunotherapy in the perioperative management of locally advanced but resectable UTUC remains undefined and requires further investigation.

Since the addition of durvalumab as an immunotherapy agent to NAC, followed by adjuvant durvalumab after cystectomy, has resulted in significantly improved event-free survival and OS in MIBC, the question arises whether these benefits may also extend to patients with UTUC [42]. To date, the only completed phase II trial with 50 patients conducted in 10 centers evaluating the safety and efficacy of a combination of immunotherapy and neoadjuvant platinum-based chemotherapy in high-risk UTUC is the iNDUCT-GETUG V08 study [43]. The trial did not meet its primary endpoint, as the ypT0 rates were 13% in the cisplatin-based combination cohort and 5% in the carboplatin-based cohort, both lower than initally hypothesized (25% and 21%, respectively). However, neoadjuvant treatment with durvalumab and platinum-based chemotherapy resulted in tumor downstaging with an increased rate of low-risk residual tumors after treatment. Notably, 5 of the 30 patients had tumors staged as ≤T1, whereas 19 patients achieved a pathological stage of ≤ypT1 after treatment with cisplatin/gemcitabine/durvalumab. No adverse events associated with immunotherapy were observed. These encouraging preliminary downstaging results are hypothesis generating and have led to the design of a phase III trial (iNDUCT-3) to further evaluate oncologic outcomes of perioperative immunotherapy combined with NAC is planned. Another ongoing phase II/III trial (NCT04628767) is currently recruiting to evaluate dose-dense MVAC +/- durvalumab and for for cisplatin-ineligible patients durvalumab with gemcitabine in UTUC.

Adjuvant immunotherapy recommendations for UTUC are stronger within clinical guidelines. Adjuvant nivolumab is approved as a monotherapy by the FDA and the European Medicines Agency (EMA) for patients with muscle-invasive urothelial carcinoma (MIUC) who have a positive PD-L1-Status (Tumor Proportion Score (TPS) ≥ 1%). The efficacy of adjuvant nivolumab in high-risk UC was analysed in CHECKMATE 274 trial (NCT02632409), a phase III, randomized, double-blind study. The trial showed improvement in DFS with nivolumab compared to placebo in the intent-to-treat (ITT) cohort (20.8 versus 10.8 months). The survival benefit was even more pronounced in patients with PD-L1 expression ≥ 1% with a 74.5% DFS rate versus 55.7% in the placebo group [44]. Recent 3-year follow-up data of this trial demonstrated continued improvement in DFS with nivolumab. However, patients with UTUC comprised only a small proportion (21%) of the study population, and therefore UTUC-specific conclusions remain exploratory and should be interpreted with caution. In an exploratory subgroup analysis of UTUC patients, nivolumab did not show any significant effect on DFS (HR 1.15; 95% CI 0.74–1.80). In particular, UTUC of the ureter had the worst outcomes with nivolumab when compared to UTUC of the renal pelvis. Given the exploratory nature and limited statistical power of this subgroup analysis, treatment recommendations for UTUC should be made with caution and are largely extrapolated from data derived from bladder cancer cohorts [45, 46]. 

Another ICI therapy that was investigated for adjuvant treatment of MIUC is pembrolizumab. The associated phase III open-label, randomized trial AMBASSADOR (NCT03244384) demonstrated a significantly improved median DFS of 29.6 months (95% CI 20.0-40.7) following adjuvant pembrolizumab treatment compared to 14.2 months (95% CI 11.0-20.2) in the control group in high-risk UC ≥ ypT2, ≥ pT3, N + or R1. However, similar to findings with nivolumab nivolumab, the therapeutic benefit of pembrolizumab was less evident in the UTUC subgroup analysis [47]. UTUC patients represented only a small subset (22.9%), limiting the statistical power of UTUC-specific conclusions. In this subgroup no significant DFS was observed (HR 1.27; 95% CI 0.74–2.18), showing similar outcomes for renal pelvis tumors and ureteral tumors. As with CHECKMATE274, the available evidence primarily originates from a bladder cancer-dominant population and the subgroup data remain insufficient to support a definitive benefit of adjuvant ICI in patients with UTUC [47].

Consequently, adjuvant therapy with nivolumab or pembrolizumab should only be considered for patients with positive PD-L1 status and UTUC with a pathological stage ≥pT3 or ≥ypT2 and/or nodal involvement and if they are ineligible for, or decline, AC or were already treated with NAC.

Given the limited efficacy of adjuvant ICI treatment, there are ongoing efforts to enhance the antitumor immune response. One promising approach involves the addition of personalized cancer vaccines to adjuvant ICI therapy. Currently, mRNA-based vaccines encoding individualized tumor neoantigens are being evaluated in clinical trials across various tumor types, such as pancreatic ductal adenocarcinoma, colorectal cancer or non-small cell lung cancer. In the context of UTUC, the randomized, double-blind, multi-site phase II clinical trial IMCODE-004 (NCT06534983) was designed to evaluate the efficacy and safety of an adjuvant mRNA neoantigen-specific vaccine in combination with nivolumab compared to nivolumab alone in patients with high-risk MIUC. However, the trial was terminated early in March 2026 due to the decreasing relevance of purely adjuvant treatment strategies, as perioperative approaches are increasingly gaining importance in the management of UC. In parallel, another phase II trial (INTerpath-005 (NCT06305767)), currently enrolling patients, aims to assess the DFS in patients with high-risk MIUC receiving adjuvant mRNA-based vaccine plus pembrolizumab versus placebo plus pembrolizumab. In this study, randomization will be stratified by ctDNA status at baseline [48]. Results of the trial are still pending. Notably, in other tumor entities such as high-risk melanoma, the combination of an mRNA-based neoantigen vaccine with pembrolizumab has already demonstrated promising results with a prolonged recurrence-free survival compared to pembrolizumab alone, suggesting a synergistic effect between mRNA vaccine and ICI [49].

Furthermore, the efficacy of ICI therapy also appears to depend on the molecular subtype of UTUC. The luminal-papillary subtype, characterized by a high frequency of FGFR3 alterations, is associated with a T-cell depleted microenvironment and consequently shows limited responsiveness to ICI. In contrast, UTUC with a basal-squamous phenotype exhibits elevated expression of immune-checkpoint molecules such as PD-L1, suggesting an enhanced response to ICI treatment. These observations underscore the importance of the immune microenvironment and molecular profile as potential predictive determinants of ICI response [50, 51]. 

## Radiotherapy

For selected cases of UTUC Radiotherapy (RT) may present an option as well, however, the overall evidence for a systemic use remains low and mostly relies on retrospective studies. A systematic review and meta-analysis of radiotherapy in an adjuvant setting after RNU stated that adjuvant RT in cases of locally advance or margin-positive disease after RNU significantly reduces the risk of locoregional recurrence with an OR of 0.43, but has not an effect on 3-year OS and 5-year CSS [52]. Nevertheless, it has to be taken into consideration that all 20 analyzed studies (ranging from 1991 to 2020) but one have been retrospective studies and case reports (*n* = 3), covered different ranges of applied radiation doses (20–66.6 Gy), were unevenly distributed considering the number of analysed patients (ranging from 21 to 2572). Moreover, study quality (according to MINORs criteria) of the analysed studies was low in 6, intermediate in 9, and high only in 2 studies (case reports 2 low, 1 high). Another systematic review identified 14 studies investigating the effects of adjuvant radiation in bladder cancer and 14 studies in UTUC after RNU [53]. Of note, certain studies are identifical to the first mentioned report, also encompassing retrospective studies with a high variance of included patients and radiation therapy regimes. Again, majorly no significant survival benefit is reporter. In addition, recently published observational retrospective studies support a role of radiotherapy in controlling local recurrence rate if chemotherapy is not an option for the patients [54, 55]. From a prospective database analysis encompassing 114 UTUC patients (final analysis including 102 patients) with isolated retroperitoneal lymph node metastasis after RNU published in 2025 the combination of RT with systemic treatment (pre enfortumab vedotin era, 2-year OS: 87.9%) demonstrated significant survival benefits over systemic therapy (2-year OS: 45.6%) or radiation therapy (2-year OS: 48.1%) alone [56]. Altogether, the evidence for the use of RT in UTUC remains sparse and relies on retrospective studies. In consequence, current guidelines on UTUC from the EAU and AUA do not recommend the systematic use of RT in locally advanced UTUC, if other options (surgery, systemic treatment) are available [1, 13].

## New drugs under investigation

Apart from established systemic approaches, several novel agents and therapeutic combinations are currently being investigated with the aim of improving oncologic outcomes and overcoming resistance to conventional treatment.

One of the agents under investigation was the fibroblast growth factor receptor (FGFR) inhibitor infigratinib. Genetic alterations in the FGFR3 occur in 21–38% of muscle-invasive UTUC and respective patients might benefit from treatment targeting FGFR receptor. In a phase Ib trial, 14 patients (including 9 with FGFR alterations) were enrolled and received preoperative treatment with infigratinib. The therapy was well tolerated and achieved a response rate of 66.7% in patients with FGFR3 alterations, with 60% of patients initially considered for RNU subsequently undergoing kidney-sparing endoscopic treatment [57]. A large phase III trial (PROOF 302) was also initiated in 2020 to evaluate infigratinib as an adjuvant 1 year treatment in patients with high-risk, muscle-invasive UC (85% UTUC) and susceptible FGFR3 alterations (NCT04197986). The studies were stopped early due to slow accrual and termination of all infigratinib oncology trials [58].

Antibody drug conjugates (ADC) with targets highly expressed in UC, such as Nectin-4, TROP2 or HER2 have shown promising single-agent activity in clinical trials and even better results when combined with ICIs, ultimately leading to the approval of enfortumab vedotin (anti-Nectin-4) in combination with pembrolizumab in metastatic UC [59]. ADC-ICI combinations might be an attractive approach also in adjuvant treatment of muscle-invasive UTUC, as shown by a retrospective study of disitamab vedotin (anti-HER2) with toripalimab (ICI), harbouring a 12-month DFS rate of 91.7% in a small sample of 12 patients [60]. Further studies are necessary to evaluate the potential benefit of ADC treatment in the perioperative treatment of locally advanced UTUC.

## Conclusion

**Fig. 1:**
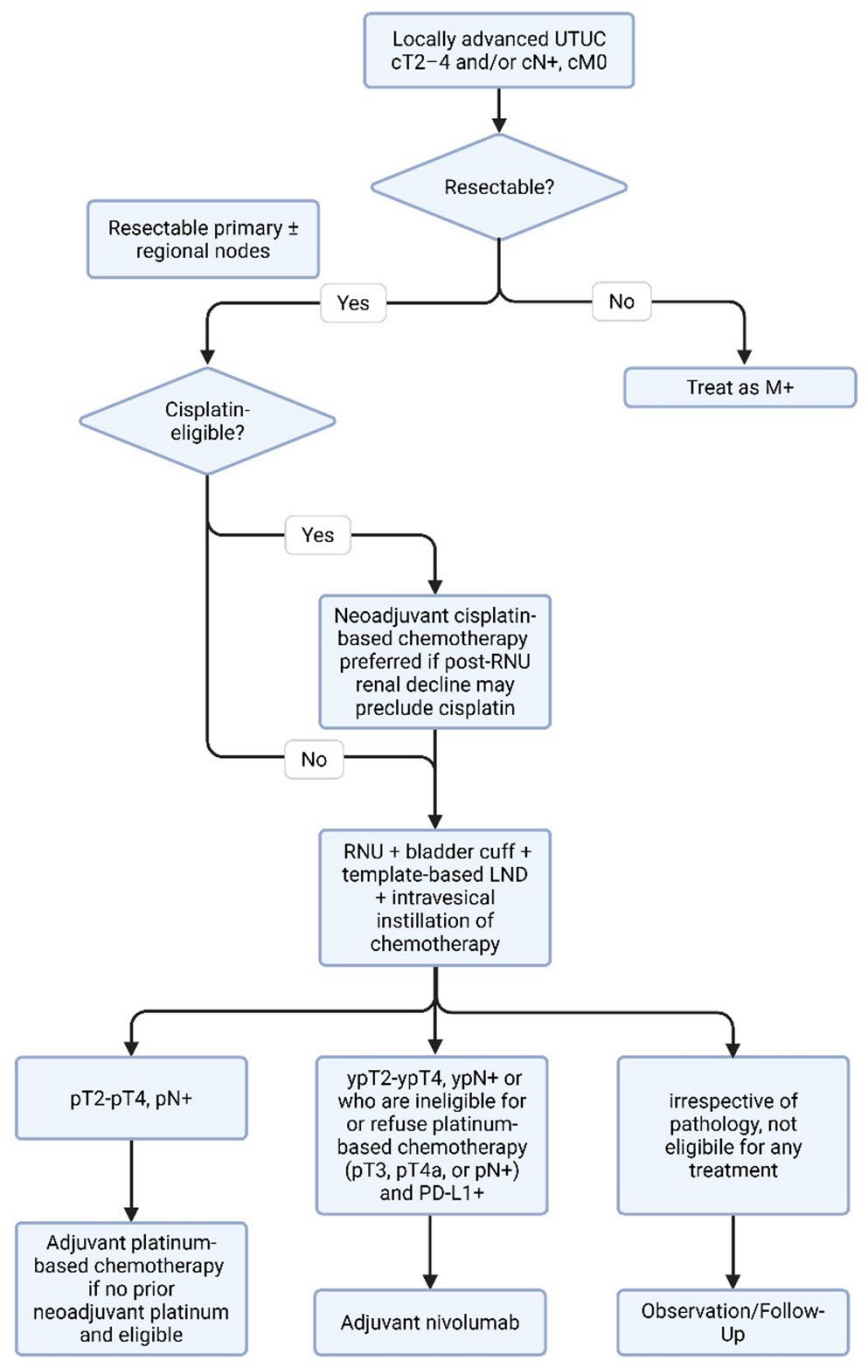
Flowchart for the Perioperative Management of UTUC (based on [13]). UTUC Upper tract urothelial carcinoma. Created with BioRender.com [61]

The management of UTUC is constantly evolving, with an increasing complexity in available treatment options (Fig. 1). Due to the heterogeneity of the tumor, shifting from one-size-fits-all strategy toward a more personalized and tailored treatment approach will be required. Importantly, treatment decisions should always be based on informed shared decision-making between physicians and patients to optimize compliance and therapeutic outcomes. The current recommendations for clinical decision-making in patients with locally advanced UTUC are given in Table 3. Despite recent therapeutic advances in the past decade, the prognosis for patients with locally advanced UTUC remains poor and more patients participating in clinical trials for this rare disease are necessary to improve the therapeutic field.

**Table 3:** Adjuvant treatment recommendations for pT2-4 and/or pN+ UTUC considering the postoperative patient status (based on [1, 13])

Cisplatin eligible	Carboplatin eligible	PD-L1 status	Treatment recommendation (always consider clinical trial as alternative, if appropriate)
Yes	Yes	Positive	3–4 cycles Gemcitabine/Cisplatin (if no prior NAC) OR Nivolumab adjuvant (if prior NAC or refusing AC)
Yes	Yes	Negative	3–4 cycles Gemcitabine/Cisplatin (if no prior NAC)
No	Yes	Positive	3–4 cycles Gemcitabine/Carboplatin (if no prior NAC) OR Nivolumab adjuvant (if prior NAC or refusing AC)
No	Yes	Negative	3–4 cycles Gemcitabine/Carboplatin (if no prior NAC)
No	No	Positive	Nivolumab adjuvant (exclude pT2 if no prior NAC)
No	No	Negative	Observation

NAC: neoadjuvant chemotherapy; AC: adjuvant chemotherapy

## Data Availability

No datasets were generated or analysed during the current study.
